# Supplementary material for: Using cellular device location data to estimate visitation to public lands: Comparing device location data to U.S. National Park Service’s visitor use statistics
Source: PLoS One. 2023 Nov 9;18(11):e0289922. doi: 10.1371/journal.pone.0289922 (PMC10635495; doi:10.1371/journal.pone.0289922)
Supplement: S1 Table — (DOCX) [file pone.0289922.s001.docx]

S1 Table. Data sources and summary statistics of total visitation and visitation by month for years 2018 & 2019 for 38 parks used in the analysis.

| **Data** | **Ground truth** | **Cell data** | **Total number of records** |
| --- | --- | --- | --- |
| **Source** | NPS Stats | Airsage Inc. |  |
| **Temporal Resolution** | Monthly | Monthly |  |
| **Total Visitation** | 25,123,3405 | 50,287,3957 | 786 |
|  |  |  |  |
| **Total Visitation By Month** |  |  |  |
| **January** | 4,847,318 | 26,856,388 | 44 |
| **February** | 8,812,831 | 32,884,283 | 69 |
| **March** | 13,588,617 | 40,668,177 | 60 |
| **April** | 21,813,190 | 39,739,348 | 69 |
| **May** | 27,245,668 | 42,081,080 | 70 |
| **June** | 33,197,005 | 49,114,604 | 72 |
| **July** | 38,241,884 | 52,074,246 | 69 |
| **August** | 34,844,865 | 52,059,138 | 73 |
| **September** | 26,348,333 | 49,318,752 | 70 |
| **October** | 20,949,009 | 47,729,356 | 69 |
| **November** | 12,901,754 | 38,918,180 | 67 |
| **December** | 8,442,931 | 31,430,404 | 54 |
|  |  |  |  |
| **Mean Visitation (standard deviation [sd]) By Month** | | | |
| **January** | 110,166 (sd = 94,655) | 610,372 (sd = 791,094) | 44 |
| **February** | 127,722 (sd = 90,618) | 476,584 (sd = 726,304) | 69 |
| **March** | 226,477 (sd = 173,916) | 677,803 (sd = 864,732) | 60 |
| **April** | 316,133 (sd = 228,048) | 575,933 (sd = 737,057) | 69 |
| **May** | 389,224 (sd = 215,119) | 601,158 (sd = 743,047) | 70 |
| **June** | 461,070 (sd = 230,031) | 682,147 (sd = 749,416) | 72 |
| **July** | 554,230 (sd = 260,933) | 754,699 (sd = 772,724) | 69 |
| **August** | 477,327 (sd = 238,802) | 713,139 (sd = 808,552) | 73 |
| **September** | 376,405 (sd = 229,874) | 704,554 (sd = 860,013) | 70 |
| **October** | 303,609 (sd = 223,335) | 691,730 (sd = 871,235) | 69 |
| **November** | 192,563 (sd = 164,564) | 580,868 (sd = 816,629) | 67 |
| **December** | 156,351 (sd = 142,786) | 582,045 (sd = 824,567) | 54 |
