# Supplementary material for: Using cellular device location data to estimate visitation to public lands: Comparing device location data to U.S. National Park Service’s visitor use statistics
Source: PLoS One. 2023 Nov 9;18(11):e0289922. doi: 10.1371/journal.pone.0289922 (PMC10635495; doi:10.1371/journal.pone.0289922)
Supplement: S2 Table — CIs stands for Confidence Intervals. (DOCX) [file pone.0289922.s002.docx]

S2 Table. Regression model for all parks combined. CIs stands for Confidence Intervals.

| *Predictors* | *Estimates* | *95% CIs* | *p* |
| --- | --- | --- | --- |
| (Intercept) | 1.848 | 1.063, 2.633 | **<0.001** |
| Cell [log] | 0.863 | 0.809, 0.917 | **<0.001** |
| January | -1.009 | -1.138, -0.881 | **<0.001** |
| February | -0.852 | -0.968, -0.737 | **<0.001** |
| March | -0.688 | -0.799, -0.577 | **<0.001** |
| April | -0.424 | -0.530, -0.318 | **<0.001** |
| May | -0.202 | -0.306, -0.098 | **<0.001** |
| June | -0.105 | -0.207, -0.002 | **0.047** |
| July | Reference |  |  |
| August | -0.048 | -0.150, 0.054 | 0.360 |
| September | -0.168 | -0.273, -0.064 | **0.002** |
| October | -0.376 | -0.481, -0.271 | **<0.001** |
| November | -0.693 | -0.805, -0.582 | **<0.001** |
| December | -0.768 | -0.889, -0.647 | **<0.001** |
|  |  |  |  |
| **Random Effects** |  |  |  |
| σ^2^ | 0.1 | | |
| τ_00_ | 1.08 _NPSCode_ | | |
| ICC | 0.92 | | |
| N | 38 _NPSCode_ | | |
| Observations | 786 | | |
| Marginal R^2^ / Conditional R^2^ | 0.555 / 0.964 | | |
