# Supplementary material for: Using cellular device location data to estimate visitation to public lands: Comparing device location data to U.S. National Park Service’s visitor use statistics
Source: PLoS One. 2023 Nov 9;18(11):e0289922. doi: 10.1371/journal.pone.0289922 (PMC10635495; doi:10.1371/journal.pone.0289922)
Supplement: S3 Table — CIs stands for Confidence Intervals. (DOCX) [file pone.0289922.s003.docx]

S3 Table. Stratified analysis by population center and iconic status. CIs stands for Confidence Intervals.

|  | **Urban & Iconic** | | | **Non-Urban & Iconic** | | | **Urban & Local** | | | **Non-Urban & Local** | | |
| --- | --- | --- | --- | --- | --- | --- | --- | --- | --- | --- | --- | --- |
| *Predictors* | *Estimates* | *95% CIs* | *p* | *Estimates* | *95% CIs* | *p* | *Estimates* | *95% CIs* | *p* | *Estimates* | *95% CIs* | *p* |
| (Intercept) | 9.094 | 8.175, 10.013 | **<0.001** | 0.159 | -0.781, 1.098 | 0.745 | 12.24 | 9.634, 14.847 | **<0.001** | 0.415 | -2.227, 3.056 | 0.767 |
| Cell [log] | 0.330 | 0.255, 0.405 | **<0.001** | 1.011 | 0.940, 1.081 | **<0.001** | 0.058 | -0.129, 0.245 | 0.561 | 0.913 | 0.725, 1.101 | **<0.001** |
| January | -0.971 | -1.164, -0.778 | **<0.001** | -0.532 | -0.731, -0.334 | **<0.001** | -1.641 | -1.875, -1.408 | **<0.001** | -1.290 | -1.587, -0.993 | **<0.001** |
| February | -0.844 | -0.953, -0.735 | **<0.001** | -0.643 | -0.821, -0.465 | **<0.001** | -1.420 | -1.648, -1.192 | **<0.001** | -1.032 | -1.318, -0.747 | **<0.001** |
| March | -0.257 | -0.354, -0.159 | **<0.001** | -0.625 | -0.787, -0.463 | **<0.001** | -0.891 | -1.111, -0.671 | **<0.001** | -0.827 | -1.110, -0.543 | **<0.001** |
| April | 0.103 | 0.016, 0.191 | **0.027** | -0.362 | -0.518, -0.207 | **<0.001** | -0.683 | -0.905, -0.461 | **<0.001** | -0.614 | -0.901, -0.326 | **<0.001** |
| May | 0.173 | 0.087, 0.259 | **<0.001** | -0.141 | -0.283, 0.002 | 0.058 | -0.473 | -0.704, -0.243 | **<0.001** | -0.355 | -0.632, -0.077 | **0.017** |
| June | 0.076 | -0.010, 0.162 | 0.095 | -0.044 | -0.181, 0.093 | 0.538 | -0.275 | -0.499, -0.050 | **0.022** | -0.208 | -0.483, 0.066 | 0.152 |
| July | Referent |  |  | Referent |  |  | Referent |  |  | Referent |  |  |
| August | -0.053 | -0.141, 0.035 | 0.254 | 0.053 | -0.083, 0.190 | 0.452 | -0.253 | -0.477, -0.029 | **0.035** | -0.188 | -0.461, 0.085 | 0.194 |
| September | -0.220 | -0.313, -0.127 | **<0.001** | 0.092 | -0.051, 0.235 | 0.217 | -0.548 | -0.768, -0.327 | **<0.001** | -0.453 | -0.728, -0.178 | **0.002** |
| October | -0.200 | -0.286, -0.113 | **<0.001** | -0.208 | -0.358, -0.059 | **0.008** | -0.621 | -0.841, -0.401 | **<0.001** | -0.595 | -0.871, -0.319 | **<0.001** |
| November | -0.460 | -0.553, -0.367 | **<0.001** | -0.626 | -0.796, -0.455 | **<0.001** | -0.890 | -1.116, -0.664 | **<0.001** | -0.967 | -1.248, -0.686 | **<0.001** |
| December | -0.578 | -0.704, -0.452 | **<0.001** | -0.631 | -0.814, -0.448 | **<0.001** | -1.105 | -1.336, -0.873 | **<0.001** | -1.106 | -1.401, -0.811 | **<0.001** |
| **Random Effects** | | | | | | | | | | | | |
| σ^2^ | 0.01 | | | 0.07 | | | 0.08 | | | 0.13 | | |
| τ_00_ | 0.08 _NPSCode_ | | | 0.14 _NPSCode_ | | | 0.13 _NPSCode_ | | | 0.54 _NPSCode_ | | |
| ICC | 0.84 | | | 0.66 | | | 0.63 | | | 0.81 | | |
| N | 8 _NPSCode_ | | | 16 _NPSCode_ | | | 7 _NPSCode_ | | | 7 _NPSCode_ | | |
| Observations | 165 | | | 314 | | | 145 | | | 162 | | |
| Marginal R^2^ / Conditional R^2^ | 0.721 / 0.954 | | | 0.880 / 0.959 | | | 0.507 / 0.820 | | | 0.659 / 0.934 | | |
