# Supplementary material for: Using cellular device location data to estimate visitation to public lands: Comparing device location data to U.S. National Park Service’s visitor use statistics
Source: PLoS One. 2023 Nov 9;18(11):e0289922. doi: 10.1371/journal.pone.0289922 (PMC10635495; doi:10.1371/journal.pone.0289922)
Supplement: S4 Table — CIs stands for Confidence Intervals. (DOCX) [file pone.0289922.s004.docx]

S4 Table. Stratified analysis by park size and porousness level. CIs stands for Confidence Intervals.

|  | Large & Low | | | | Large & High | | | Small & Low | | | Small & High | | |
| --- | --- | --- | --- | --- | --- | --- | --- | --- | --- | --- | --- | --- | --- |
| *Predictors* | *Estimates* | *95% CIs* | | *p* | *Estimates* | *95% CIs* | *p* | *Estimates* | *95% CIs* | *p* | *Estimates* | *95% CIs* | *p* |
| (Intercept) | 0.076 | -0.990, 1.141 | | 0.892 | 0.706 | -1.245, 2.656 | 0.49 | 9.545 | 7.664, 11.426 | **<0.001** | 8.114 | 6.888, 9.340 | **<0.001** |
| Cell [log] | 1.024 | 0.943, 1.106 | | **<0.001** | 0.883 | 0.748, 1.018 | **<0.001** | 0.285 | 0.142, 0.427 | **<0.001** | 0.402 | 0.305, 0.500 | **<0.001** |
| January | -0.514 | -0.753, -0.275 | | **<0.001** | -1.079 | -1.282, -0.876 | **<0.001** | -1.742 | -1.988, -1.495 | **<0.001** | -1.223 | -1.404, -1.041 | **<0.001** |
| February | -0.583 | -0.793, -0.372 | | **<0.001** | -1.107 | -1.305, -0.909 | **<0.001** | -1.177 | -1.383, -0.970 | **<0.001** | -0.839 | -0.988, -0.689 | **<0.001** |
| March | -0.649 | -0.843, -0.455 | | **<0.001** | -0.792 | -0.981, -0.603 | **<0.001** | -0.637 | -0.833, -0.441 | **<0.001** | -0.364 | -0.510, -0.218 | **<0.001** |
| April | -0.317 | -0.503, -0.131 | | **0.001** | -0.627 | -0.820, -0.434 | **<0.001** | -0.321 | -0.506, -0.136 | **0.001** | 0.006 | -0.127, 0.140 | 0.929 |
| May | -0.091 | -0.264, 0.082 | | 0.315 | -0.394 | -0.577, -0.212 | **<0.001** | -0.195 | -0.387, -0.004 | 0.055 | 0.119 | -0.013, 0.252 | 0.096 |
| June | -0.027 | -0.196, 0.143 | | 0.763 | -0.182 | -0.361, -0.003 | 0.053 | -0.155 | -0.339, 0.030 | 0.114 | 0.043 | -0.090, 0.175 | 0.55 |
| July | Referent |  | |  | Referent |  |  | Referent |  |  | Referent |  |  |
| August | 0.075 | -0.093, 0.243 | | 0.392 | -0.199 | -0.377, -0.020 | **0.034** | -0.175 | -0.362, 0.011 | 0.076 | -0.052 | -0.186, 0.082 | 0.47 |
| September | 0.079 | -0.097, 0.255 | | 0.39 | -0.388 | -0.565, -0.211 | **<0.001** | -0.454 | -0.645, -0.264 | **<0.001** | -0.192 | -0.330, -0.054 | **0.011** |
| October | -0.212 | -0.397, -0.027 | | **0.028** | -0.581 | -0.759, -0.402 | **<0.001** | -0.418 | -0.605, -0.232 | **<0.001** | -0.243 | -0.376, -0.110 | **0.001** |
| November | -0.615 | -0.822, -0.409 | | **<0.001** | -0.934 | -1.122, -0.747 | **<0.001** | -0.735 | -0.929, -0.542 | **<0.001** | -0.434 | -0.573, -0.295 | **<0.001** |
| December | -0.591 | -0.812, -0.370 | | **<0.001** | -1.09 | -1.286, -0.893 | **<0.001** | -0.884 | -1.110, -0.659 | **<0.001** | -0.616 | -0.776, -0.455 | **<0.001** |
|  | | |  |  |  |  |  |  |  |  |  |  |  |
| **Random Effect** |  | | | |  | | |  | | |  | | |
| σ^2^ | 0.09 | | | | 0.08 | | | 0.07 | | | 0.02 | | |
| τ_00_ | 0.12 _NPSCode_ | | | | 0.55 _NPSCode_ | | | 0.40 _NPSCode_ | | | 0.10 _NPSCode_ | | |
| ICC | 0.57 | | | | 0.87 | | | 0.85 | | | 0.82 | | |
| N | 14 _NPSCode_ | | | | 11 _NPSCode_ | | | 8 _NPSCode_ | | | 5 _NPSCode_ | | |
| Observations | 280 | | | | 225 | | | 173 | | | 108 | | |
| Marginal R^2^ / Conditional R^2^ | 0.853 / 0.937 | | | | 0.600 / 0.949 | | | 0.396 / 0.911 | | | 0.743 / 0.954 | | |
