# Supplementary figures and images for: Using cellular device location data to estimate visitation to public lands: Comparing device location data to U.S. National Park Service’s visitor use statistics
Source: PLoS One. 2023 Nov 9;18(11):e0289922. doi: 10.1371/journal.pone.0289922 (PMC10635495; doi:10.1371/journal.pone.0289922)

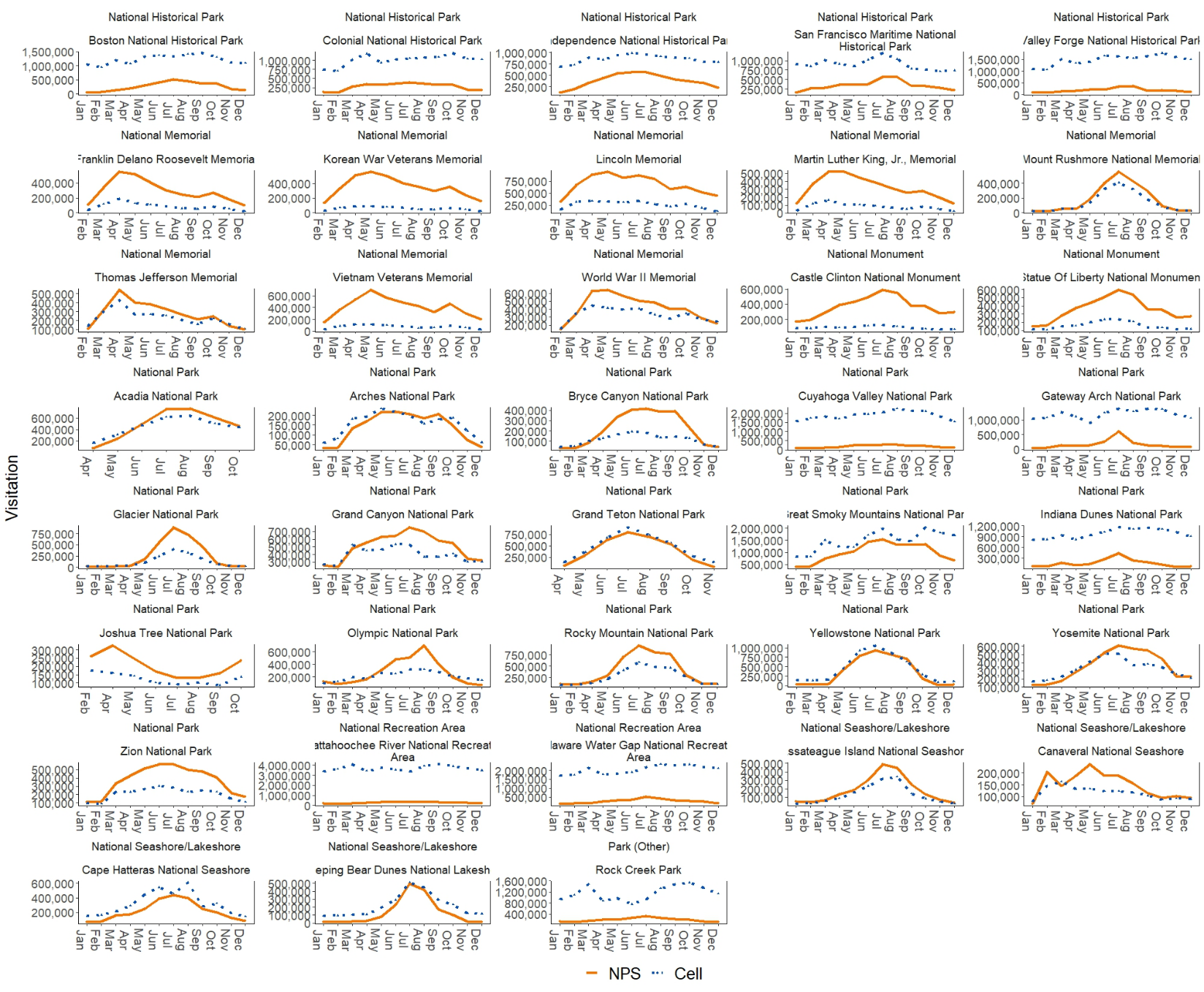


S1 Fig. Average monthly visitation using NPS counts and cell data for each park for years 2018 and 2019.

Supplement: S1 Fig — (DOCX) [file pone.0289922.s005.docx]
